# Development and Analytical Evaluation of a Point-of-Care Electrochemical Biosensor for Rapid and Accurate SARS-CoV-2 Detection

**DOI:** 10.3390/s23188000

**Published:** 2023-09-20

**Authors:** Mesfin Meshesha, Anik Sardar, Ruchi Supekar, Lopamudra Bhattacharjee, Soumyo Chatterjee, Nyancy Halder, Kallol Mohanta, Tarun Kanti Bhattacharyya, Biplab Pal

**Affiliations:** 1 Department of Virology, Opteev Technologies Inc., Baltimore, MD 21225, USA; bpal@prophecycorp.com; 2 Research and Development Laboratory, Opteev Healthtech, GN-4, Sector-V, Kolkata 700091, India; asardar@prophecycorp.com (A.S.); rsupekar@opteevresearch.com (R.S.); lbhattacharjee@prophecycorp.com (L.B.); sochatterjee@opteevresearch.com (S.C.); nhalder@prophecycorp.com (N.H.); kmohanta@prophecycorp.com (K.M.); 3 Department of Electronics and Electrical Communication Engineering, Indian Institute of Technology, Kharagpur 721302, India; tkb@ece.iitkgp.ac.in

**Keywords:** SARS-CoV-2, electrochemical biosensor, lactam-stapled peptide, impedance spectroscopy

## Abstract

The COVID-19 pandemic has underscored the critical need for rapid and accurate screening and diagnostic methods for potential respiratory viruses. Existing COVID-19 diagnostic approaches face limitations either in terms of turnaround time or accuracy. In this study, we present an electrochemical biosensor that offers nearly instantaneous and precise SARS-CoV-2 detection, suitable for point-of-care and environmental monitoring applications. The biosensor employs a stapled hACE-2 N-terminal alpha helix peptide to functionalize an in situ grown polypyrrole conductive polymer on a nitrocellulose membrane backbone through a chemical process. We assessed the biosensor’s analytical performance using heat-inactivated omicron and delta variants of the SARS-CoV-2 virus in artificial saliva (AS) and nasal swab (NS) samples diluted in a strong ionic solution, as well as clinical specimens with known Ct values. Virus identification was achieved through electrochemical impedance spectroscopy (EIS) and frequency analyses. The assay demonstrated a limit of detection (LoD) of 40 TCID_50_/mL, with 95% sensitivity and 100% specificity. Notably, the biosensor exhibited no cross-reactivity when tested against the influenza virus. The entire testing process using the biosensor takes less than a minute. In summary, our biosensor exhibits promising potential in the battle against pandemic respiratory viruses, offering a platform for the development of rapid, compact, portable, and point-of-care devices capable of multiplexing various viruses. The biosensor has the capacity to significantly bolster our readiness and response to future viral outbreaks.

## 1. Introduction

Recent years have witnessed an escalating global concern over respiratory virus outbreaks [1,2]. The emergence of severe acute respiratory syndrome coronavirus 2 (SARS-CoV-2) and the ensuing COVID-19 pandemic have underscored the critical need for rapid and accurate screening, diagnostics, and treatment strategies for virus-related diseases [3,4]. While significant progress has been made in developing effective COVID-19 vaccines [5], rapid and dependable virus detection remains pivotal in addressing future respiratory viral epidemic surges [6].

Numerous approaches have been employed to detect respiratory viruses, notably the prominent SARS-CoV-2. These methodologies primarily encompass nucleic acid detection techniques, such as reverse transcription polymerase chain reaction (RT-PCR)-based methods, and point-of-care (PoC) lateral flow assays [7,8,9]. While real-time PCR is renowned for its outstanding precision and specificity, its intricate procedure results in delayed reporting and requires specialized equipment and expertise [10,11]. Conversely, PoC methods like rapid antigenic tests (RATs) offer relatively swift results, albeit with some compromise in accuracy [12]. There is a pressing need for alternative approaches that are sensitive, cost-effective, rapid, and amenable to mass production for both point-of-care and self-screening applications.

In this regard, electrochemical biosensor technology emerges as a highly promising solution due to its high sensitivity, selectivity, cost-effectiveness, and fast response [13,14]. Electrochemical biosensors incorporate bioreceptors affixed to the surface of the working electrode, facilitating direct and specific binding with target molecules that subsequently generate quantifiable signals for analysis [15]. In recent years, a plethora of electrochemical biosensors have been developed for the detection of various pathogens and cancer biomarkers [16,17,18]. However, the efforts in this domain have experienced a significant surge in the last three years, primarily attributed to the COVID-19 pandemic. In this context, several noteworthy advancements have been achieved. For instance, Guojun et al. [19] devised an electrical biosensor using graphene-field-effect transistors (G-FETs) to detect RNA from COVID-19 patients. By employing a single-stranded DNA probe on the graphene electrode surface that binds to viral RNA, they achieved high sensitivity, with a limit of detection as low as ~0.1 fg mL^−1^. Similarly, Ghumra et al. recently introduced a portable biosensor for SARS-CoV-2 detection in exhaled breath, utilizing spike-protein-specific nanobodies on a micro-immunoelectrode. The biosensor detects as few as 10 viral particles per sample through tyrosine amino acid oxidation [20]. Detailed reviews of current advances in electrochemical biosensor development are presented in reviews by Patel et al. and Samson et al. [21,22]. A detailed comparison of recent efforts in the development of biosensors and conventional approaches is shown in Table 1.

One of the major challenges in the development and commercialization of electrochemical biosensors pertains to the selection of materials suitable for mass production that can maintain reproducibility and stability under diverse environmental conditions. This choice of materials directly impacts the limit of detection, susceptibility to non-specific adsorption of interfering substances, and the sensor’s overall reproducibility and stability when applied in complex real-world matrices [23]. For instance, the utilization of modified nanomaterial surfaces has been explored to achieve exceedingly low LoDs [24,25], but often with issues of reproducibility due to challenges in controlling the synthesis and immobilization of nanoparticles, leading to variations in size, shape, conformation, and topology between sensors [23,26]. Various materials, including gold nanoparticles, diverse carbon forms (such as graphene, graphene oxides, and carbon nanotubes), metal oxides, and conductive polymers (CPs), have been investigated for electrode construction in electrochemical biosensors [27,28,29,30]. Among these materials, CPs, characterized by their unique π orbital structure and conformational changes, exhibit superior sensitivity, selectivity for specific biological molecules, and rapid electrical responses in biosensors [27,31,32]. Furthermore, CP properties can be easily tailored through functionalization or monomer coupling, leading to enhancements in electronic properties and sensor stability [27]. In addition to the sensor material, immobilizing biomolecule probes (e.g., aptamers, antibodies, ssDNA, synthetic peptides) on the electrode surface is crucial for sensor performance [33,34]. Among these biorecognition molecules, peptides, resembling proteins in selectivity and specificity but being smaller and more stable, are excellent bioreceptor alternatives in biosensing due to cost-effectiveness, ease of modification, and enhanced chemical versatility [35,36].

CP-based electrochemical biosensors often employ electrochemical impedance spectroscopy (EIS), ideal for point-of-care use due to simplicity, high sensitivity, and speed. EIS finds applications in diverse biorecognition tasks, such as lipid bilayer monitoring [37], DNA testing [38], detecting small biological molecules [39], and cancer diagnosis [40]. A recent study reported the development of a SARS-CoV-2 biosensor using screen-printed gold electrodes functionalized with thiolated synthetic peptides, allowing direct electrochemical impedance spectroscopy monitoring of their interaction with spike proteins. The platform demonstrated notable sensitivity and reproducibility, with a detection limit of 18.2 ng/mL for spike protein in commercial solutions and 0.01 copies/mL for lysed SARS-CoV-2 particles. However, the assay has a 15 min turnaround time [35]. In our opinion, the utilization of CP-modified electrodes in conjunction with synthetic peptide functionalization and EIS analysis effectively tackles several common challenges encountered by electrochemical biosensors. These include enhancing assay sensitivity, reducing response time, and improving selectivity by minimizing background signals.

**Figure 1:**
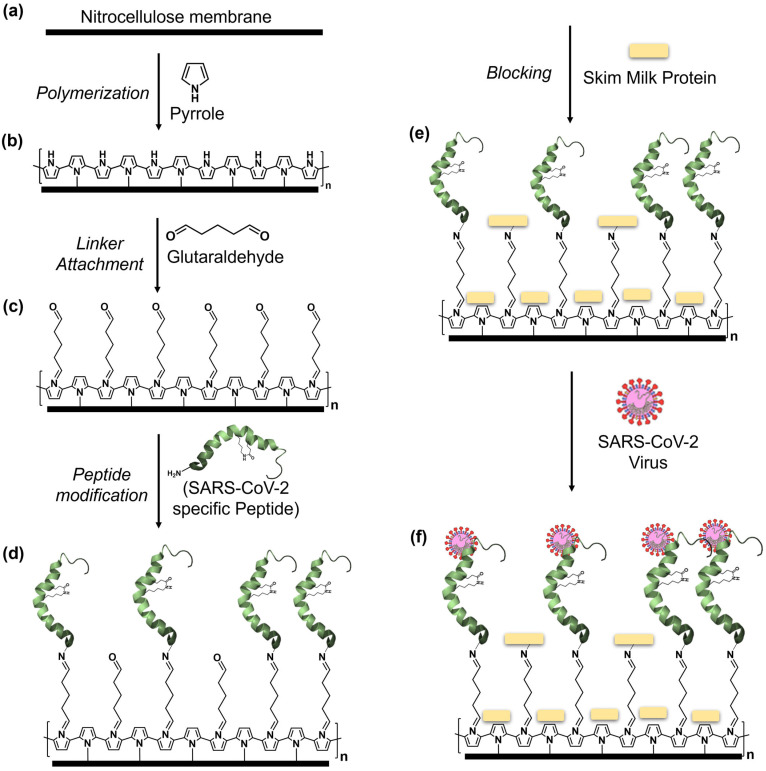
Schematic presentation of biosensor development. (**a**) Nitrocellulose membrane (NC) (1 mm × 10 mm in dimension) as the base of the sensor substrate, (**b**) polymerization of conducting polymer (polypyrrole) on NC membrane, (**c**) covalent attachment of organic linker (glutaraldehyde), (**d**) functionalization with lactam stapled SARS-CoV-2 specific peptide, (**e**) blocking with skim milk protein, and (**f**) interaction of the SARS-CoV-2 virus with the receptor peptide.

This study introduces a conducting polymer-based biosensor meticulously designed for the specific detection of the SARS-CoV-2 virus. The fundamental conducting material, polypyrrole, is integrated onto a nitrocellulose membrane backbone, drawing on its established role in biosensing. Leveraging bio-receptor interaction, we achieved selective and stable biosensor functionality by coupling lactam-stapled hACE2 receptor-based peptides using a glutaraldehyde linker. The choice of the hACE2 α1-helix as the immobilizing biomolecule is informed by computational and experimental studies, showing its potential to inhibit the receptor binding domain (RBD)-hACE2 complex formation and subsequent host cell infection as a therapeutic approach [41,42,43]. However, alpha 1-helix-based hACE2 peptides were reported to lose bioactivity in solution, affecting RBD binding [44,45]. To address this issue, we adopted a lactam i, i+4 stapling modification described by Maas et al. and Nevola et al. [46,47] to stabilize the hACE-2 peptide structure. A schematic illustration of the design and workflow of the biosensor development is shown in Figure 1. To assess analytical performance, the biosensor was tested using heat-inactivated omicron and delta variants of the SARS-CoV-2 virus spiked into artificial saliva and nasal swabs, the latter suspended in high ionic solution, as well as using stored clinical nasal swabs with known Ct values. Sensitivity, specificity, and the limit of detection were evaluated, comparing the biosensor’s performance with published rapid antigen test data and other similar biosensors. Notably, this biosensor incorporates a robust electrical signal data analysis strategy, meticulously optimizing the frequency range in impedance measurement. This strategic refinement effectively neutralizes the influence of noise that could compromise accuracy. This algorithmic augmentation ensures precise and reliable virus detection, thereby enhancing the overall efficacy and durability of the biosensor system. 

**Table 1:** Summary of different avenues towards detection of SARS-CoV-2 virus.

Platforms		Analyte	Sensitivity and LoD	Response Time	Advantages	Refs.
Laboratory based						
PCR		Nucleic Acid	98–100% and 10–100 copies/mL	3–4 h	High specificity High sensitivity Accurate estimation of viral load	[48,49,50,51]
ELISA		Antigen, Antibodies	85–90% and 0.01–0.1 ng	1–5 h	Low LoD Simple procedure	[52,53,54]
Point of Care (POCs)						
RATs		Antigen	60–70% and 10–100 PFU/mL	13–15 min	Fast response Cost effective	[55,56,57]
Biosensors	Electrical	Nucleic Acid Antigen	High and 0.1–1 fg/mL	5–10 min	Low LoD High sensitivity	[19]
	Optical	Antibody	86.7% and <2 ng/spot	<30 min	Very high specificity Low LoD	[58]
	Optical	Nucleic Acid	97.5% and 10 ng/mL	30 min	Low LoD High sensitivity	[37]
	Optical	Antigen	High and 100 copies/mL	<15 min	High accuracy	[59]
	Opto-magnetic	Nucleic Acid	10 copies/µL and 0.4 fM	100 min	Low LoD High Sensitivity	[60]
	Electrochemical	N-gene	231 copies/µL and <10 copies/µL	<5 min	Low LoD High sensitivity Fast response	[61]
	Electrochemical	Reactive oxygen species	97% and <500 µL	<30 s	Fast detection High accuracy	[62]
	Electrochemical	Spike Protein	77.8% and 20–30 copies/mL	<1 min	Non-invasive High sensitivity Fast response Low cost	[20]
	Electrochemical	Spike Protein	High and 18.2 ng/mL	15 min	Label free High sensitivity High reproducibility	[35].
	Electrochemical	Antigen	95% and 40 TCID50/mL	1 min	Low LoD High sensitivity Fast response Non-invasive	This Work

## 2. Materials and Methods

### 2.1. Reagents

Pyrrole (C_4_H_5_N) 98.0% (Merck, Darmstadt, Germany) was vacuum-distilled before use. Ammonium persulphate ((NH_4_)_2_S_2_O_8_) 98.0% (APS) and glutaraldehyde (OHC(CH_2_)_3_CHO) (Grade 1, 25% in water) were obtained from Merck, Darmstadt, Germany. Nitrocellulose membrane (pore size 0.45 µm) was purchased from Fisher Scientific. All other chemicals were of analytical grade and used without further purification. Hydrochloric acid (HCl), phosphate-buffered saline (PBS. pH 7.4), and de-ionized water were obtained from Merck (Darmstadt, Germany), SRL (Mumbai, India), and Emplura (Merck, Darmstadt, Germany), respectively. The hACE2 peptide sequence (Figure S1) used in this study was adopted from Maas et al. [46] and was purchased from GL Biochem (Shanghai) Ltd. (Shanghai, China) The peptide sequence specifically binds to the RBD region of the SARS-CoV-2 spike protein. The lactam-linked stapling modification on the peptide at positions K12 and E20 (i, i+4) enhances the stability of its three-dimensional structure. For the peptide–virus binding evaluation experiments, the peptide was tagged with the Alexa Fluor 488 NHS ester (green) dye (A270022, antibodies.com, Cambridge, UK).

### 2.2. Viruses and Clinical Specimens

The viruses utilized in this study were SARS-CoV-2 lineage B.1.617.2 (Delta Variant) culture fluid (heat-inactivated, 0810624CFHI, Zeptometrix LLC, Buffalo, NY, USA) and SARS-CoV-2 lineage B.1.1.529 (Omicron Variant) culture fluid (UV-inactivated, 0810642UV, Zeptometrix LLC, Buffalo, NY, USA) and (heat-inactivated, 0810642CFHI, Zeptometrix LLC, Buffalo, NY, USA). For control experiments, Influenza vaccine (Fluarix-Tetra 2021 South, GlaxoSmithKline Biologicals, Munich, Germany) containing an attenuated mix of Influenza A and B viruses (A/Victoria/2570/2019 (H1N1), A/Hong Kong/2671/2019 (H3N2), B/Washington/02/2019 and B/Phuket/3037/2013) was used. We directly employed the pre-inactivation viral titers provided by the manufacturer in TCID_50_/mL, which can be found in the product inserts for detailed reference. Additionally, we quantified the virus stocks through RT-PCR analysis using a standard curve generated with the 10-fold serially diluted positive control (provided with the kit) of the known copy number using the Coronavirus COVID-19 Genesig real-time PCR assay kit (Z-Path-COVID-19-CE, Primer Design Ltd., Eastleigh, UK) (Figure S2). To evaluate the sensitivity and specificity of the biosensor, the virus, originally present in culture supernatant, was subjected to buffer exchange into the desired media (Artificial saliva, SAE0149, Sigma-Aldrich, St. Louis, MO, USA). The buffer exchange process was conducted using Nanosep 3K Omega columns (OD003C33, Pall corporations). During the experimental procedure of detecting viruses, 1 µL of freshly prepared viral solution was dropped on the biosensor using a micropipette and impedance measurement was conducted. To assess the biosensor’s performance in real matrices, frozen clinical samples from nasopharyngeal swabs, collected from COVID-19-positive patients, were provided by the Centre for Clinical Research at the John C. Martin Centre for Liver Research and Innovation, Indian Institute of Liver & Digestive Sciences. These samples, obtained as part of the “SARS-CoV-2 breath analyzer Diagnostic Study” and confirmed through RT-PCR testing, were preserved in universal transport medium (UTM). Seven distinct specimens with varying Ct values (representing low, medium, and high Cts) were specifically chosen and subjected to testing using the biosensor.

### 2.3. Development of the Biosensors

Development of the biosensors on the nitrocellulose substrate was carried out following a sequential approach as shown in Figure 1. Initially, specific dimensions of nitrocellulose membranes (1 mm × 10 mm) were coated with polypyrrole via oxidative polymerization of pyrrole monomer in an acidic medium using ammonium persulfate (APS) as the oxidizing agent. A mixture of 0.1 M pyrrole and 0.1 M APS in 1 M HCl was used, and the substrates were immersed in this solution (1:1 ratio) at 10 °C for in situ polypyrrole deposition, which took around 120 min. The resulting polypyrrole-coated substrates were rinsed with deionized water and air-dried. Following substrate preparation, a linker, glutaraldehyde, was covalently attached by treating the polypyrrole-coated substrates with 25% glutaraldehyde for 4 h, facilitating flexible bridges for effective binding of biomolecules [63,64]. After washing with phosphate-buffered saline (PBS), site-specific SARS-CoV-2 RBD lactam-stapled hACE-2 peptide was covalently immobilized onto the glutaraldehyde-treated substrates at 30 µg/mL concentration in 1× PBS for 16 h. Excess and unbound peptide were removed by washing with 1× Tris-buffered saline with Tween20 (TBST). To reduce non-specific binding, 5% skim milk in 1× PBS was used to block binding sites for 90 min. After further washing with 1× TBST and deionized water, the substrates were vacuum-dried for about 2 h, serving as the basis for biosensor fabrication.

### 2.4. Assessment of Selective hACE-2 Peptide Binding to SARS-CoV-2

To confirm the specific binding of the hACE-2-stapled peptide to the SARS-CoV-2 virus, we conducted a fluorescence imaging study. Ppy-coated glass slides (5 mm × 5 mm) were immersed in a 30 µg/mL peptide solution prepared in 1× PBS for 16 h on a rocking shaker. Following this, the glass slides were washed with 1× TBST buffer and then treated with 5% skimmed milk for 90 min to block nonspecific binding sites. After another TBST wash, the glass slides were exposed to the virus for one hour. Specifically, SARS-CoV-2 delta and omicron variants served as peptide-specific controls, while an Influenza Vaccine containing a mix of Influenza A and B virus variants (as described in the ‘Viruses’ section) was used as a peptide non-specific control. A skimmed-milk-treated glass slide without virus exposure served as the negative control. Following a 1× TBST wash, the glass substrates were treated with Alexa fluor 488 (green dye) NHS-ester-tagged peptide for 30 min. Subsequently, the substrates were rinsed with 1× TBST and left to dry overnight in darkness. Fluorescence images of the glass slides were captured using a GFP filter at 10× magnification.

### 2.5. Electrochemical Impedance Measurement

In the experimental phase, the sensor strips were affixed to electrical connectors at both ends using silver paste. These connected sensors were then inserted into an impedance analyzer, specifically the PGSTAT204 potentiostat from Autolab (Berlin, Germany), for virus detection experiments. As a two-electrode sensor configuration was employed, the potentiostat’s electrode arrangement adheres to the designated scheme (Figure S3), involving a working electrode biased relative to the reference electrode without a ground connection, while the source and counter electrodes serve to facilitate charge flow. An alternating current (AC) bias of 100 mV was applied, and the frequency was scanned logarithmically from 20 kHz to 40 kHz with 10 points per decade. The initial step entails measuring the impedance of a blank sensor. Subsequently, 1 μL of either virus or control solution sample was dispensed onto the sensor’s central region. Within 5–10 s of sample exposure, another impedance measurement was initiated. The first scan of the sensor (either blank or dry) was denoted as the initial impedance, while the scan following sample exposure was termed the final impedance. The entire frequency scan duration for each set of measurements (initial and final impedance) was approximately 20 s. Given that significant impedance changes are primarily observed in the magnitude upon sample exposure, the analysis focuses solely on the magnitude of impedance. To account for potential variations in the initial impedance values among sensors from different batches, a normalized impedance change parameter (dZ/Z) is introduced using the formula:

dZZ=1−ZfZi

where dZ represents the change in total impedance post-sample exposure, Z_f_ denotes the final impedance after one minute of sample exposure, and Z_i_ signifies the initial impedance of the sensor. Notably, this parameter’s value is also influenced by the probing frequency. The impedance analysis was carried out within a specific frequency range of 1 kHz to 100 kHz, chosen and fine-tuned through empirical optimization.

## 3. Results and Discussion

**Figure 2:**
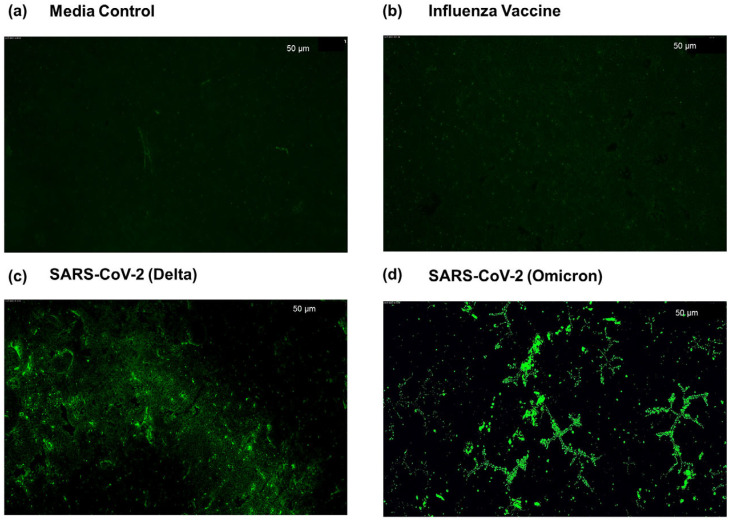
Selective binding of SARS-CoV-2 to a lactam-based stapled hACE-2 functionalized on Ppy substrate on glass slide. The Ppy-coated and glutaraldehyde-linked glass slides were treated with hACE-2 peptide and blocked with skim milk protein before addition of virus or controls. Alexa Fluor 488 fluorophore-tagged peptide was then used to probe virus binding. (**a**) Artificial saliva without virus spike-in was used as a media control. (**b**) Heat-attenuated influenza vaccine containing a mix of Influenza A (H1N1, H3N2) and B viruses. (**c**) SARS-CoV-2 delta variant at concentration of 10^5^ virus copies/µL. (**d**) SARS-CoV-2 omicron variant at concentration of 10^5^ virus copies/µL.

As described in the Materials and Methods section, we assembled the biosensor by polymerizing Ppy on NC membrane backbone and functionalizing it with the 35-mer lactam-based i, i+4-stapled hACE2 N-terminal α1-helix inhibitor 1 sequence as outlined by Maas et al. [46], with the aim of establishing a rapid SARS-CoV-2 detection method. To evaluate the virus’s selective binding to this peptide, we initiated the process by coating a glass slide with Ppy, introducing a glutaraldehyde linker, and functionalizing the biosensor with the stapled hACE-2 peptide at a concentration of 30 μg/mL. Subsequently, a 5% skim milk protein solution was used to block non-specific binding on the sensor. Next, the SARS-CoV-2 omicron variant virus, at a concentration of 3 log_10_TCID_50_/mL in artificial saliva, was applied to the biosensor and incubated for one hour at room temperature. Following a washing step, the biosensor was exposed to an Alexa 488-tagged hACE-2 peptide to interact with the virus–biosensor complex. After additional washing with 1× TBST, images were captured via fluorescence microscopy. To ascertain specificity, a heat-inactivated influenza virus was used as a control. As anticipated, the biosensor demonstrated selective binding to the SARS-CoV-2 virus, exhibiting no binding to the Influenza viruses, even at a high concentration of 120 µg/mL of haemagglutinin (HA) protein, thereby affirming no cross-reactivity (Figure 2).

### 3.1. Characterization of Biosensor and Detection of SARS-CoV-2 Using Electrochemical Impedance Spectroscopy

**Figure 3:**
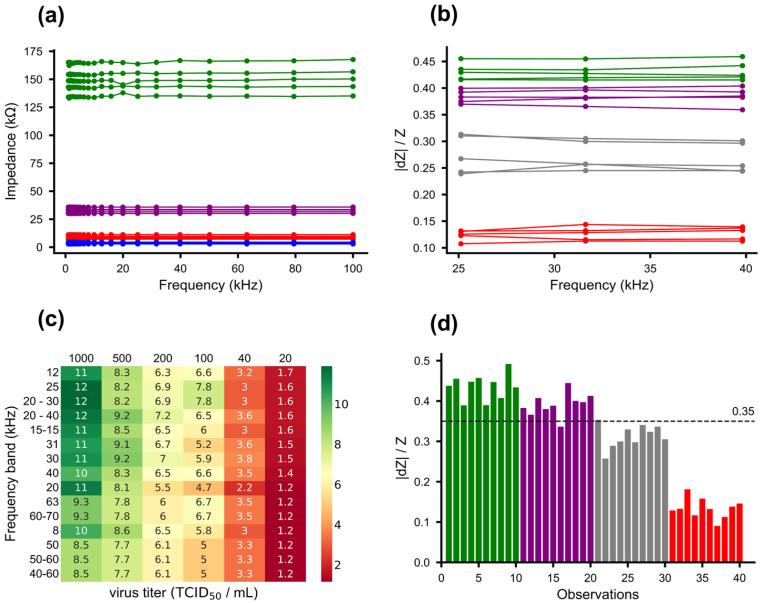
Characterization of biosensor and detection of SARS-CoV-2 virus using electrochemical impedance spectroscopy. (**a**) Characteristic impedance measurement at different stages of sensor development across a range of frequencies. Blue lines: after coating with polypyrrole, red lines: after GA linker addition, purple lines: after hACE2 peptide attachment, and green lines: after blocking with skimmed milk protein. (**b**) Sensor response in terms of relative impedance change (|dZ|/Z) for different virus concentrations and media control. Green lines are media control; purple, grey, and red lines are virus in artificial saliva at concentrations of 20, 40, and 1000 TCID_50_/mL, respectively. (**c**) Heatmap for optimization of separation factor between virus and control class. Frequency band corresponds to the optimum separation factor (sf) between media control and different virus concentrations. The greener shades indicate higher sf values while the reddish shades indicate lower sf values. The rows are sorted in descending order based on the sf values of viral RNA copies 20 TCID_50_/mL. (**d**) Sensor response in terms of relative impedance change (|dZ|/Z) for different virus concentrations and media control (10 representative samples from 20 replicates) separated by a threshold line. Green bars are media control; purple, grey, and red bars are viral RNA copies (20 TCID_50_/mL, 40 TCID_50_/mL, and 1000 TCID_50_/mL, respectively). A dashed black threshold line is drawn at 3 standard deviations below the mean of the control data set.

As previously mentioned, EIS is a widely recognized technique for characterizing impedimetric biosensors and analyzing interfacial properties associated with bio-recognition events [65]. In line with EIS’s suitability, we conducted impedance measurements (details provided in the Materials and Methods section) at various stages of sensor development to ensure fabrication process reproducibility and establish baseline impedance levels for each biosensor development step. As depicted in Figure 3a, distinct bands of absolute impedance were evident after addition of each component on the nitrocellulose membrane substrate, with steadily increasing impedance. Polypyrrole functions as a *p*-type semiconductor where holes are the dominant charge carriers [66]. The incremental impedance rise suggests a gradual depletion of charge carriers due to the negative charges of entities such as glutaraldehyde, peptides, and skim milk protein binding to Ppy. These negative entities attract positive holes from the conducting polymer backbone, diminishing the carrier count and thus elevating total impedance [67].

Subsequently, impedance alterations were gauged upon the introduction of the quantified virus. Specifically, 1 µL of SARS-CoV-2 virus was added to the biosensor in five independent replicates, with high concentration (1000 TCID_50_/mL) and two lower concentrations (40 and 20 TCID_50_/mL), and impedance measurements were conducted (Figure 3b). As a negative control, 1 µL of artificial saliva was applied to offset impedance shifts arising from charges within the virus media. The outcomes unveiled distinctive impedance bands corresponding to virus concentration on the biosensor, with the most pronounced distinction between virus data (highlighted by red lines in Figure 3b) and control data (emphasized by green lines in Figure 3b) evident at the highest virus concentration (1000 TCID_50_/mL). Nevertheless, the data also exhibited fluctuations and noise, particularly within the lower frequency ranges.

To mitigate the impact of noise, we embarked on data pre-processing strategies with the goal of identifying the optimal frequency or frequency range that maximizes the discernment between the control and virus classes, particularly for lower virus concentrations such as 20 and 40 TCID_50_/mL. Initially, the sensor’s responses to virus and control samples were recorded in terms of the modulus of impedance change, represented by the alteration in impedance (dZ) from its initial value (Z) before the introduction of the virus or control droplet. Subsequently, segregation of control data from various virus concentration data was achieved using the 3-sigma rule principle [68], assuming normal data distribution. Figure S4a,b in the Supplementary Materials showcase the characteristic normal distribution for the 40 TCID_50_/mL virus concentration, alongside a normality test. Leveraging the assumed normal distribution of other data points, we computed the separation factor (sf) between the virus and control classes using the following formula

sf=μcontrol−μvirusσcontrol+σvirus

where sf denotes the separation factor, μ_control_ and μ_virus_ represent the means of the control and virus data, respectively, while σ_control_ and σ_virus_ represent their respective standard deviations.

The separation factor between the control and varying virus concentrations was computed across individual frequencies and a range of frequency bands. Subsequently, frequencies were sorted based on descending sf values specifically for the lowest virus concentration (20 TCID_50_/mL), leading to the creation of a heatmap (Figure 3c). A higher separation factor denotes a more pronounced distinction between the virus and control classes. In this study, a separation factor of 3 indicates strong capability, signifying a high likelihood of distinguishing the virus from the control or an error probability of 0.27% in virus detection. Similarly, a separation factor of 1.96 serves as a cutoff, representing a 95% probability of separation or a 5% error probability in virus detection. As shown in Figure 3c, sf values are depicted for both control and diverse virus concentrations. Notably, the heatmap highlights a peak separation factor at 12 kHz for the 20 TCID_50_/mL virus concentration. However, within the 20 kHz to 40 kHz frequency band, consistently higher separation factors are evident across most virus concentrations. This initial analysis was used to determine the preliminary limit of detection of the biosensor (see detail below).

### 3.2. Determination of Virus Detection Based on Relative Impedance Threshold

As stated earlier, impedance spectroscopy is a well reported approach to characterize biosensors towards detection of viruses and bacteria, but such reports are mostly focused on relative impedance measurement [35,69,70]. To determine the classification of individual sample tests as positive or negative virus detection, we devised a comprehensive threshold line computation strategy based on the principle of Gaussian likelihood [71], encompassing distinct steps. First, the optimal input frequency was pinpointed as outlined previously. Subsequently, the central tendency of the relative impedance (dZ/Z) values from control data corresponding to the chosen frequency band for each test data was calculated, utilizing the median as the central tendency metric. The mean (μ_control_) and standard deviation (σ_control_) were then computed across all control test data. Then, a decisive threshold line was established based on the following equation:

$$Thresholdline=\mu_{control}-\left(3\times \sigma_{control}\right)$$

Responses below this established threshold line were identified as belonging to the virus class, whereas those exceeding it were categorized as control class. The specified threshold, set at 0.35, notably exhibited exceptional sensitivity of 95% in distinguishing the 40 TCID_50_/mL virus concentration data from control data, as shown in Figure 3d (with 10 representative samples from each virus concentration among the 20 independent replicates). Alternatively, a machine learning (ML) approach was adopted to determine the threshold value to validate the results obtained from the Gaussian likelihood method. The use of machine learning to determine threshold value has been previously reported by Li et al., who showed that the use of a decision-tree-based threshold reduces the false detection from 36% to 7% [72]. Similarly, we have used a decision tree to generate the threshold value, based on the normalized impedance change data from the biosensor. The implementation of decision trees is reported by Friedman et al. [73] in various applications. Our approach involves the selection of features for the model, the segregation of the dataset into training and test data, the choice of an appropriate model, model training, model evaluation, threshold selection, and model validation.

Following a parallel methodology, an optimal frequency range was selected for data processing, with |dZ|/Z values serving as pivotal feature variables for the model. The dataset, constituting 40 test data from both control (n = 20) and virus experiments (n = 20), was randomly divided into a 75:25 ratio to form distinct training and testing datasets. A decision tree model of depth 1 was fitted, culminating in a threshold value of approximately 0.37 (Figure S5). This threshold was subsequently tested on the designated dataset, yielding an impressive 100% accuracy in discerning 40 TCID_50_/mL virus concentration data from control data. Furthermore, the established threshold from the decision tree model was rigorously validated using a completely new dataset comprising 25 virus test samples and 25 control test samples, yielding remarkable outcomes of 100% sensitivity. Detailed validation results are presented in the Supplementary Materials (Figure S5). However, in adherence to a conservative approach and due to the limited dataset for the machine learning model, we retained the earlier formula derived based on the Gaussian likelihood principle for drawing threshold lines.

### 3.3. Limit of Detection, Sensitivity, and Specificity of the Biosensor

**Figure 4:**
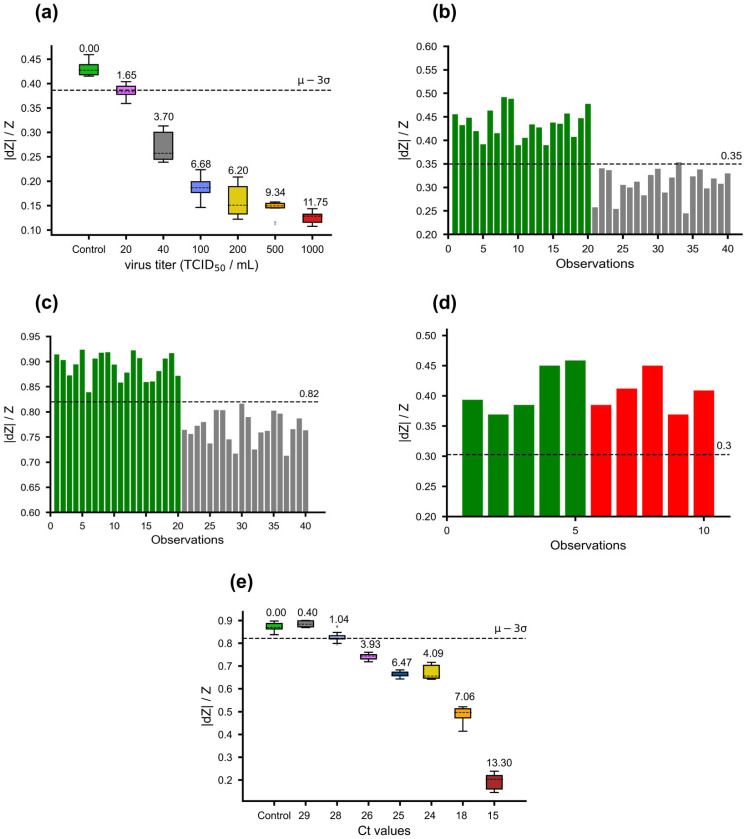
Classification of virus detection: (**a**) Relative impedance change (|dZ|/Z) for different virus concentrations and media control. The colors indicate virus concentrations: green (media control), purple (20 TCID_50_/mL), grey (40 TCID_50_/mL), blue (100 TCID_50_/mL), yellow (200 TCID_50_/mL), orange (500 TCID_50_/mL), and red (1000 TCID_50_/mL). sf values are annotated for each box. (**b**) Limit of detection validation at 40 TCID_50_/mL. Y-axis: relative impedance value (|dZ|/Z). Green and grey bars represent media control and virus, respectively. (**c**) Comparable sensitivity of virus spiked in nasal swabs in 0.45 M KCl buffer. Y-axis: relative impedance value (|dZ|/Z). Green and grey bars represent media control and virus, respectively. (**d**) Relative impedance changes for influenza vaccine and media control. y-axis: relative impedance value (|dZ|/Z). Green and red bars represent media control and influenza, respectively. (**e**) Evaluation of frozen clinical specimens. X-axis represents Ct values from RT-PCR experiments. Each sample is tested in five replicates. The colors of boxes correspond to the different Ct values. sf values are annotated for each box. Threshold lines are denoted by black dashed lines for all figures and represent 3 standard deviations below the mean of control data’s relative impedance change.

To comprehensively evaluate our biosensor’s analytical performance, we conducted tests to establish the limit of detection, sensitivity, and specificity. Initially, we performed a 10-fold assessment to determine preliminary LoD (Figure S6) followed by testing selected concentrations from a 2-fold serially diluted virus analyte in artificial saliva (Figure 4a). Integrating these tests with our previous analyses (refer to Figure 3b,d), we identified the concentration yielding a minimal yet above-the-cut-off separation factor (1.96) as the preliminary LoD. Consequently, we set the assay’s detection limit at 40 TCID_50_/mL virus concentration, affirming this determination through 20 replicates at the specified LoD, in accordance with the requirements of the Emergency Use Authorization program of the Food and Drug Administration (FDA EUA), aiming for 95% sensitivity. In parallel, 20 replicates of artificial saliva without virus were analyzed. As depicted in Figure 4b, the assay exhibited remarkable sensitivity at this LoD, detecting 19 positives out of 20 replicates and accurately identifying all negatives, providing 95% sensitivity and 100% specificity. Furthermore, considering that many COVID tests utilize nasal swab specimens, we acquired nasal swabs from healthy donors suspended in a high ionic buffer (0.45 M KCl) and spiked with equal virus concentration (40 TCID_50_/mL) for assessment with our biosensor. Notably, the results showcased similar sensitivity to that observed with virus in artificial saliva (Figure 4c). There was no notable difference in sensitivity between the omicron and delta variants of SARS-CoV-2 in sensitivity (Figure S7). Furthermore, the biosensor was tested with heat-attenuated influenza vaccine containing a mix of Influenza A (H1N1, H3N2) and B viruses and did not show any cross-reactivity (Figure 4d).

Finally, we assessed our biosensor’s performance in clinical matrices obtained from the “SARS-CoV-2 breath analyzer Diagnostic Study,” conducted by the Centre for Clinical Research at the John C. Martin Centre for Liver Research and Innovation, Indian Institute of Liver & Digestive Sciences. Seven nasopharyngeal swabs, collected in UTM and frozen as leftovers, were chosen based on their RT-PCR Ct values and categorized into low (Cts 17 and 18)-, medium (Cts 26, 27, 28)-, and high (Cts 30 and 31)-Ct-value groups. Prior to biosensor testing, the samples were diluted in KCl solution to a final concentration of 0.45 M. Our biosensor successfully detected the low- and medium-Ct-value samples but had a a slight margin of error in detecting the high-Ct-value specimens (Figure 4e). We hypothesize that the biosensor’s challenge with high Ct values may be attributed to potential sample degradation, as RT-PCR can still detect fragmented RNA in these cases.

The determined 95% LoD threshold of our assay is 40 TCID_50_/mL as determined by the manufacturer’s pre-inactivation analysis. To facilitate fair comparisons with other studies evaluating RAT analytical performance, we determined viral RNA copies corresponding to dilutions of TCID_50_/mL concentrations provided by the manufacturer using RT-PCR (Figure S8). Based on this analysis, our 95% LoD threshold corresponds to 6.6 log10 RNA copies/mL. Studies on the analytical performance of RATs are diverse due to differences in study design and methods, sample size, and type of RAT evaluated, which demonstrated variable performance, and sometimes with reports of contradicting results for similar assays, making comparisons difficult [74,75,76]. Furthermore, variability in viral quantification, such as TCID_50_, plaque-forming units (pfu), or RT-PCR-based viral RNA copies, introduce bias to make comparisons [77]. However, within this context and limitations, our biosensor demonstrates comparable or better analytical performance with commonly used rapid antigen tests. For example, Corman et al. evaluated the 95% LoD of seven antigen point-of-care tests using 138 clinical specimens and reported LoDs ranging between 6.32 log_10_ and 7.46 log_10_ RNA copies per swab [78]. Similarly, Deerain et al. [76] evaluated the analytical sensitivity of 10 commercially available RATs using representative delta and omicron isolates cultured from clinical samples and reported 95% LoDs of 6.50 log_10_ copies/mL and 6.39 log_10_ copies/mL for delta and omicron variants, respectively. Put together, our data indicate that our biosensor has comparable and in some cases better analytical performance with commercially available RATs and some reported efforts with electrochemical, electrical, and optical biosensors (Table 1). While acknowledging that our developed biosensor, akin to commercially available rapid antigen tests, may not achieve the same sensitivity levels as RT-PCR, its distinct advantage lies in its rapid detection capabilities. This advantage is especially beneficial for identifying individuals with elevated viral loads, which significantly contribute to transmission dynamics. This attribute makes our biosensor a valuable tool for clinical and public health applications, particularly in various settings where swift and efficient detection is essential. Furthermore, in contrast to numerous biosensors on various platforms, our biosensor exhibits significant advantages in overall performance, including sensitivity, selectivity, response time, LoD, and stability, when exposed to clinical matrix testing. These advantages can be primarily attributed to the use of a conductive polymer as the base material for biosensor development, the employment of modified synthetic peptide as an immobilizing bioreceptor, and the utilization of EIS analysis. Table 1 provides a summary of diverse SARS-CoV-2 detection methods, encompassing both conventional approaches and various biosensors.

An additional noteworthy feature of our biosensor is its potential for heightened sensitivity through miniaturization. Currently at 1 mm x 10 mm dimension, it can be further reduced to 50 μm × 25 μm via photolithography, enhancing sensitivity by minimizing the conducting surface area, thus detecting extremely low virus concentrations, and improving its limit of detection (LoD). This sensitivity improvement is rooted in the reduced dimensions of the active binding region [50], optimizing portability, sensitivity, and overall performance while reducing power consumption compared to conventional designs [79]. Miniaturization also facilitates more efficient mass transport, faster analyte binding, and superior multiplexing capabilities for various applications [80], yet challenges like functionalization and parallel reading of closely spaced sensor locations must be tackled [81].

## 4. Conclusions

In summary, our study has successfully developed a rapid biosensor tailored for precise SARS-CoV-2 detection. This was achieved by functionalizing a conductive polymer biosensor with a lactam-stapled hACE-2 N-terminal alpha helix peptide, endowing it with exceptional selectivity. This selectivity was corroborated through both fluorescence microscopy and impedance measurement, enabling accurate virus detection in minuscule volumes of artificial saliva and nasal swabs. The biosensor demonstrated robust analytical performance, boasting 95% sensitivity and 100% specificity at a LoD of 40 TCID_50_/mL. These results are comparable to, and in many cases superior to, commercial rapid antigen tests. Subsequent clinical evaluation is necessary to assess its practical utility. Furthermore, our biosensor showcased rapid and precise SARS-CoV-2 detection, yielding results in under a minute. A noteworthy advantage of our biosensor lies in its potential for heightened sensitivity through miniaturization. This enhancement translates into improved portability for point-of-care applications, reduced power consumption, accelerated response times, and the capability to simultaneously detect multiple analytes. These attributes render our biosensor suitable for diverse applications in medical diagnostics and environmental monitoring.

## 5. Patents

A provisional patent application for the invention described in this paper was filed with the United States Patent and Trademark Office on 7 November 2023. The application number is 63/513,007.

## Data Availability

Not applicable.

## Supplementary Materials

The following supporting information can be downloaded at: https://www.mdpi.com/article/10.3390/s23188000/s1, Figure S1: Lactam-stapled hACE-2 peptide sequence and design for functionalizing biosensor; Figure S2: Standard curve generated for RT-PCR quantification of viral stocks; Figure S3: Connection configuration of potentiostat to the biosensor (green strip); Figure S4: Normality test of distribution of relative impedance change of the biosensor; Figure S5: Threshold line generated using machine learning model for detection of virus; Figure S6: Preliminary assessment of limit of detection; Figure S7. Measurement of relative impedance change using SARS-CoV-2 delta variant. Figure S8: Correlation of TCID50/mL and viral RNA copies/mL for SARS-CoV-2 delta and omicron variants.
