# Supplementary material for: Development and Analytical Evaluation of a Point-of-Care Electrochemical Biosensor for Rapid and Accurate SARS-CoV-2 Detection
Source: Sensors (Basel). 2023 Sep 20;23(18):8000. doi: 10.3390/s23188000 (PMC10534802; doi:10.3390/s23188000)
Supplement: Supplementary file 1 [file sensors-23-08000-s001.zip › sensors-2592479-supplementary.pdf]

sensors MDPI

Supplementary Materials

Development and Analytical Evaluation of a Point-of-Care Electrochemical Biosensor for Rapid and Accurate SARS-CoV-2 Detection

Mesfin Meshesha, Anik Sardar, Ruchi Supekar, Lopamudra Bhattacharjee, Soumyo Chatterjee, Nyancy Halder, Kallol Mohanta, Tarun Kanti Bhattacharyya and Biplab Pal

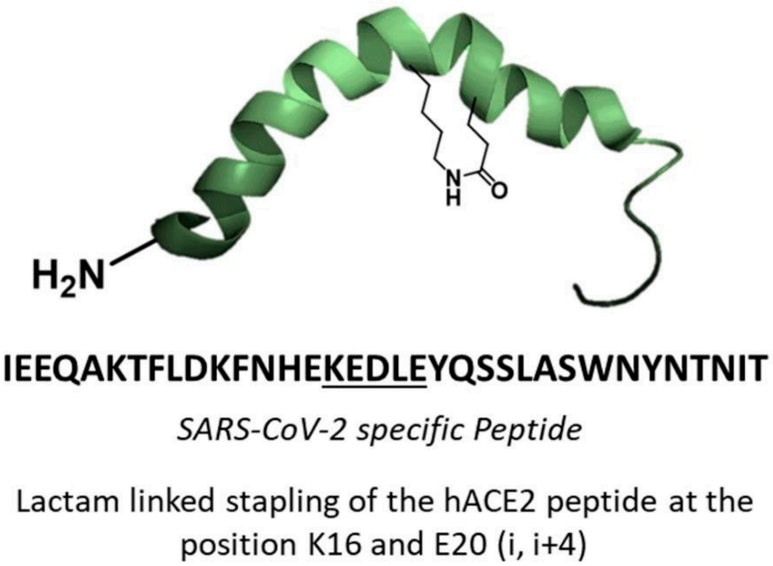

Figure S1. Lactam-based i, i + 4 stapled hACE-2 peptide sequence and design for functionalizing biosensor. The sequence and image were adopted from Maas et.al [46].

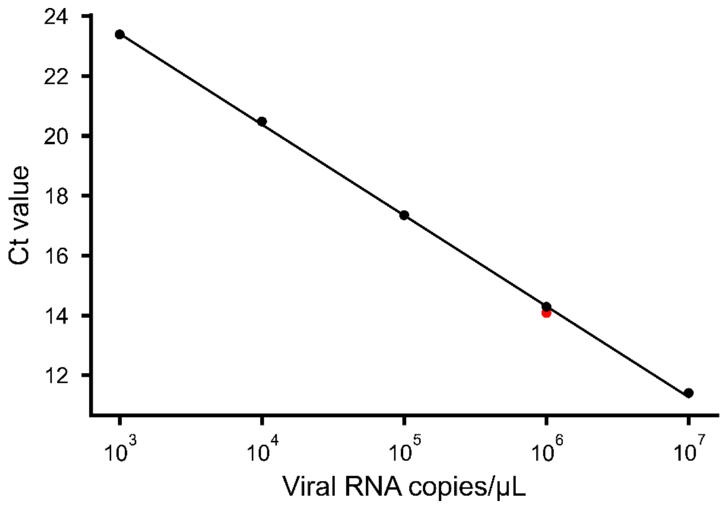

Figure S2. Standard curve generated for RT-PCR quantification of viral stocks. The standard curve is generated using the 10-fold serially diluted SARS-CoV-2 positive control of the known copy number provided with the Coronavirus COVID 19 Genesig real time PCR assay kit. The red dot on the curve indicates unknown sample concentration.

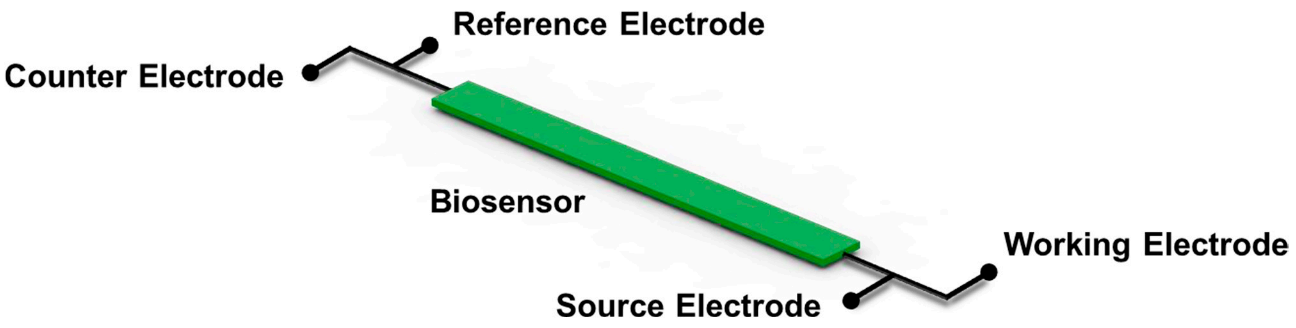

Figure S3. Connection configuration of potentiostat to the biosensor (green strip). To convert the standard set-up into a two-electrode system the working and source electrodes are coupled together, while counter and reference electrodes are shorted.

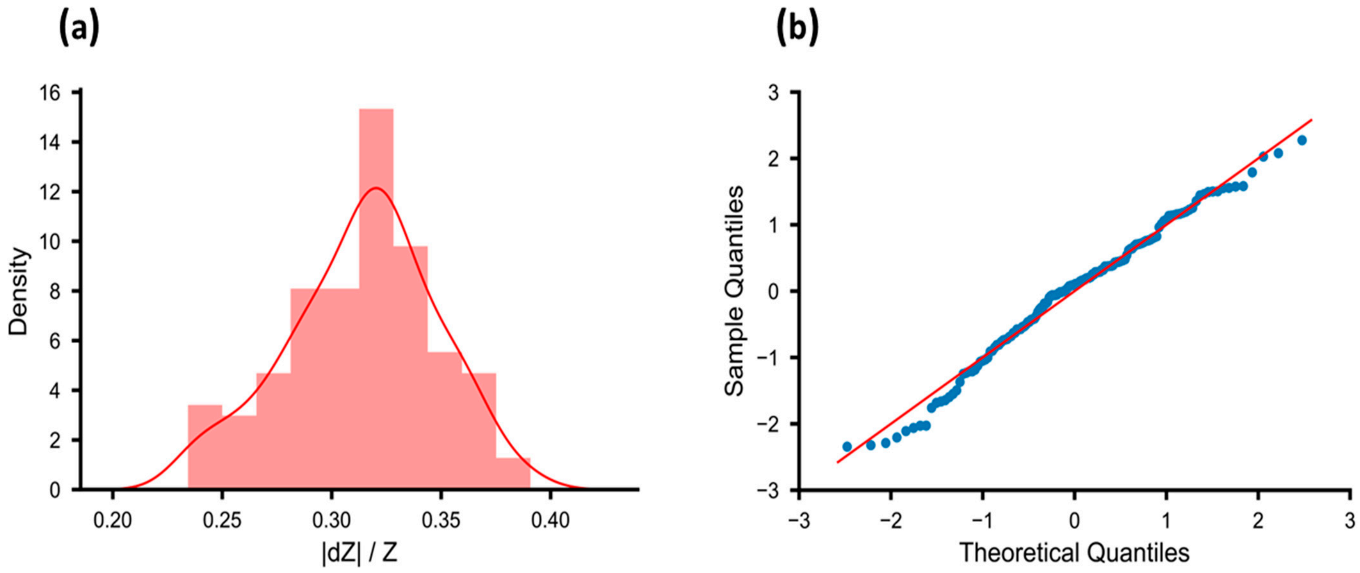

Figure S4. Normality test of distribution of relative impedance change of the biosensor. (**a**) Distribution of relative impedance (|dZ|/Z) change, (**b**) Q-Q plot of |dZ|/Z for 40 $TCID_{50}$/mL from 50 independent experiments.

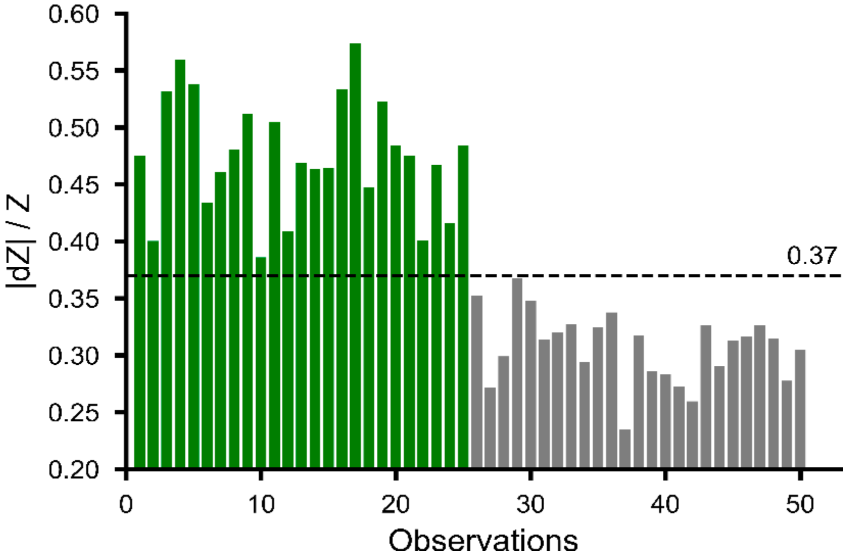

Figure S5. Threshold line generated using machine learning model for detection of virus. Y-axis indicates relative impedance value (|dZ|/Z) and X-axis represent independent tests. Green and grey bars represent media control and virus (4 $TCID_{50}$/mL) respectively. The dotted black line represents threshold line for positive and negative classification of individual tests.

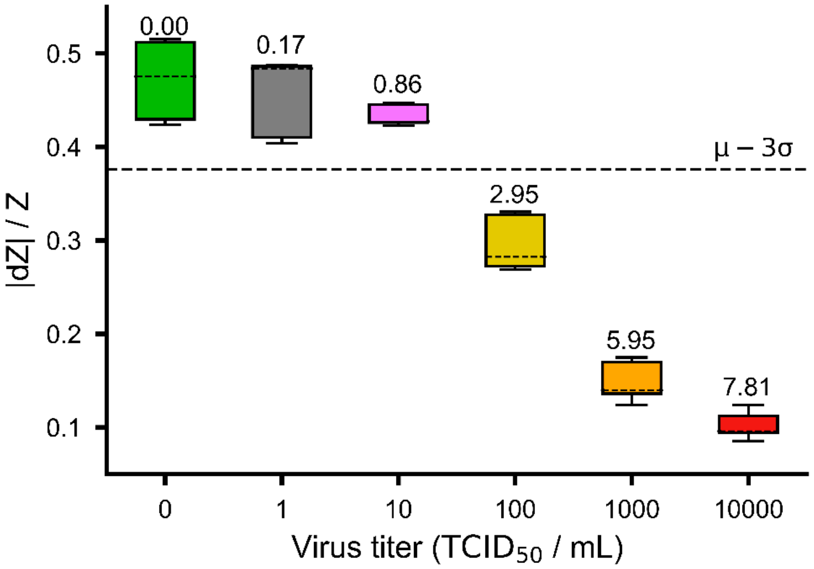

Figure S6. Preliminary limit of detection assessment: Measurement of relative impedance change (|dZ|/Z) was conducted across various 10-fold virus concentrations, accompanied by a media control. In the visualization, the media control is distinguished by the green box. Boxes shaded in varying tones of Grey, Purple, Yellow, Orange, and Red signify different virus concentrations, corresponding to viral RNA copies of 0.1 $TCID_{50}$/mL, 1 $TCID_{50}$/mL, 10 $TCID_{50}$/mL, 100 $TCID_{50}$/mL, and 1000 $TCID_{50}$/mL, respectively.

The corresponding sf values are provided as annotations above each box.

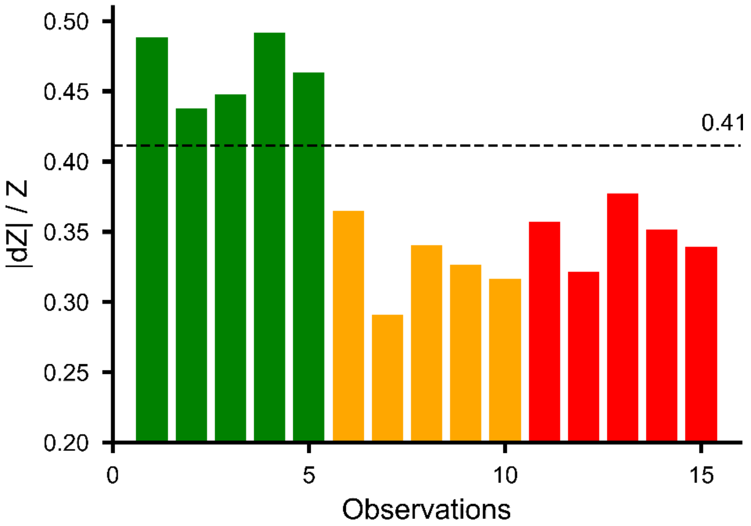

Figure S7. Measurement of relative impedance change using SARS-CoV-2 delta variant. Y-axis indicates relative impedance value (|dZ|/Z). Green, orange and red bars represent media control, Omicron variant (40 $TCID_{50}$/mL) and Delta variant (40 $TCID_{50}$/mL) respectively. The dashed black line represents the threshold line for classification of individual tests as positive or negative.

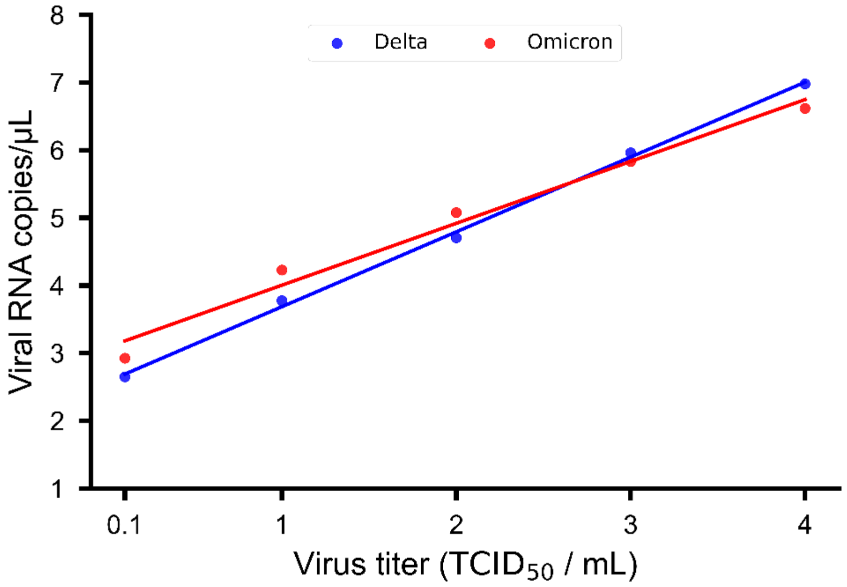

Figure S8. Correlation between $TCID_{50}$/mL and viral RNA copies/ μL for SARS-CoV-2 delta and omicron variants. The RNA copy quantification of each 10-fold $TCID_{50}$/mL dilution is determined through RT-PCR utilizing a standard curve. Both axes are presented in a log_{10} scale.
